# Psychometric properties of the 21-item Depression, Anxiety, and Stress Scale (DASS-21) among Malaysians during COVID-19: a methodological study

**DOI:** 10.1057/s41599-022-01229-x

**Published:** 2022-06-29

**Authors:** Arulmani Thiyagarajan, Tyler G. James, Roy Rillera Marzo

**Affiliations:** 1 Department of Clinical Epidemiology, Leibniz Institute for Prevention Research and Epidemiology—BIPS, Bremen, Germany; 2 Department of Family Medicine, University of Michigan, Ann Arbor, MI USA; 3 Department of Community Medicine, International Medical School, Management and Science University, Shah Alam, Malaysia; 4 Department of Community Medicine, Faculty of Medicine, Asia Metropolitan University, Johor, Malaysia; 5 Global Public Health Jeffrey Cheah School of Medicine and Health Sciences, Monash University, Subang Jaya, Malaysia

**Keywords:** Medical humanities, Psychology

## Abstract

Depression, anxiety, and stress continue to be among the largest burdens of disease, globally. The Depression, Anxiety, and Stress Scale-21 Items (DASS-21) is a shortened version of DASS-41 developed to measure these mental health conditions. The DASS-41 has strong evidence of validity and reliability in multiple contexts. However, the DASS-21, and the resulting item properties, has been explored less in terms of modern test theories. One such theory is Item Response Theory (IRT), and we use IRT models to explore latent item and person traits of each DASS-21 sub-scale among people living in Malaysia. Specifically, we aimed to assess Classical Test Theory and IRT properties including dimensionality, internal consistency (reliability), and item-level properties. We conducted a web-based cross-sectional study and sent link-based questionnaires to people aged 18 and above in a private university and requested to roll out the link. Overall and individual sub-scales’ Cronbach’s alpha of the DASS-21 indicates an excellent internal consistency. The average inter-item correlation and corrected inter-item correlations for each of the sub-scales indicated acceptable discrimination. On average, DASS-21 total scores and sub-scale scores were significantly higher among female participants than males. The Graded Response Model had better empirical fit to sub-scale response data. Raw summated and latent (IRT estimated) scores of the Depression, Anxiety, and Stress sub-scales, and overall DASS-21 were strongly correlated. Thus, this study provides evidence of validity supporting the use of the DASS-21 as a mental health screening tool among Malaysians. Specifically, standard error of measurement was minimized to provide robust evidence of potential utility in identifying participants who are and are not experiencing these mental health issues. Additional research is warranted to ensure that test content culturally appropriate and accurately measuring cultural norms of depression, anxiety, and stress.

## Introduction

Over the past decade, mental health conditions have gained attention due to their increasing burden worldwide. The 2019 Global Burden of Disease study estimates that depressive and anxiety disorders are two of the leading causes of disability globally (13th and 24th, respectively, among all causes of disability) (Vos et al., 2020). These conditions cost 1 trillion US dollars annually across the global economy (WHO, 2016). Therefore, expanding mental health services, as well as diagnosing and treating these mental health conditions, is critical for improving global health and achieving international health objectives.

In low- and middle-income countries, the lack of sustainable care, stigma, and the low awareness of mental health disorders are major factors contributing to their higher burden (Rathod et al., 2017; Wainberg et al., 2017). Malaysia is a tropical country in Southeast Asia with over 31 million population residing as per 2018 estimates (WorldBank, 2018). Though the country has improved economically over the past 50 years, the number of mental health care facilities and psychiatrists are insufficient (Parameshvara Deva, 2004). Similar to neighboring developing countries (Mia and Griffiths, 2021), the prevalence of mental health conditions in Malaysia is alarming and requires sustainable mental health care solutions (IHME, 2017). One-fifth of primary care patients in Malaysia present with anxiety or depression (Deva, 2005), and depressive disorders are the 7th highest cause of death and disability in Malaysia (IHME, 2017). However, when considering the lack of resources and health care professionals in Malaysia, the current estimate likely underrepresents the true burden of mental health issues among Malaysians.

Adequate screening and diagnosis are essential to develop treatment plans and improve patient health outcomes. While clinical judgment is always warranted, a screening tool (e.g., a rating scale) helps to have a quick indication of cross-sectional mental wellbeing and can be valuable in assisting with diagnosis and treatment. Self-reported questionnaires and clinician rated scales are common approaches to measure mental health disorders. One such self-reported questionnaire/scale is the 21-item Depression, Anxiety, and Stress Scale (i.e., DASS-21), a shortened version of DASS-41 developed to capture the constructs of depression, anxiety, and stress. The construction of the DASS-21 was based heavily on the strong evidence of reliability and validity of scores elicited from the DASS-41 (Lovibond and Lovibond, 1995). The DASS-21 is one of the most widely used depression screening measures, in addition to the Patient Health Questionnaire (9-item, 8-item, and 2-item versions), and has been used across cultures and populations (Peters et al., 2021). It not only covers the core symptoms of depression and anxiety, it was proven to discriminate well between the sub-scales (Beaufort et al., 2017). Other studies have investigated the psychometric properties across different settings and in different populations (Wardenaar et al., 2018; Yohannes et al., 2019; Lee and Kim, 2022). In Malaysian population, a few studies have explored the psychometric properties of DASS-21 using Classic Test Theory (CTT) (Musa, et al., 2007; Ramli et al., 2009), but the DASS-21 scale and its depression, anxiety, and stress sub-scales are least explored through the use of modern test theory, specifically item response theory (IRT).

IRT, or latent response theory, employs mathematical models to measure a relationship between latent traits (e.g., depression) and observed response outcomes (Hambleton et al., 1991). In CTT, the test (or in this case, entire DASS-21) is the unit of analysis. In contrast, IRT considers items as the unit of analysis, and an item’s measurement precision depends on the latent trait of a respondent (known as “theta”, *θ*). Unlike CTT, IRT is considered a strong assumption model, requiring testing of monotonicity, unidimensionality, and local independence. When these assumptions are supported, IRT allows us to examine both invariant item statistics (item-information, item-characteristics, conditional reliability) and ability estimates (discriminatory ability, location parameter, slope parameter, and overall scale characteristics) (Embretson and Reise, 2000; Fan and Sun, 2013).

The DASS-21 comprises ordinal multiple response category items; therefore, it is essential to consider the ordinality and polytomous nature of the scale items. Among different models of IRT, we use the graded response model (GRM), graded rating scale model (GRSM), and generalized partial credit model (GPCM). GRM is one of the IRT families of mathematical models for ordinal responses (Samejima, 1997). The GRM is a generalization of the two-parameter logistic (2PL) model, which estimates the probability of receiving a certain score or higher, given the level of the underlying latent trait (Keller, 2005). The 2PL model is used for dichotomous response data, while the GRM for ordered polytomous categorized data. The GRM allows us to examine the probability of a participant selecting a specific response category for each item; to estimate the test subject’s or latent trait (e.g., levels of depression); to estimate how well the test questions measure that latent trait or ability. The GRSM is a version of modified GRM that allows for a common threshold across all items. It also functions similar to GRM in terms of classical IRT parametrization, except that the graded ratings scale model applies to the slope intercept (Muraki, 1990). It assumes equivalent spacing structure across items in representing the Likert-type items. The GPCM is also same as the GRM, but the GPCM estimates separate response parameters for each category while the GRM assumes response category threshold equivalence across items. In contrast to GRM, the GPCM uses an adjacent categories approach where the probability of selecting a specific response category is not necessarily ordered; that is, for example, a response category for a higher level of depression may actually have a higher probability of selection.

We aim to use statistics estimated under the CTT paradigm and IRT models to explore the various latent traits of overall DASS-21 scale and its sub-scales’ properties. The specific objectives of our study were to evaluate the DASS-21 sub-scales using IRT, and to determine the internal consistency, factor structure, discriminating characteristics, and item-level properties.

## Methods

### Study setting

Owing to the COVID-19 pandemic, we implemented snowball sampling (convenience) recruitment methods; in our case, we disseminated the survey link (containing the questionnaire) through various digital platforms (WhatsApp, Facebook, and email) and requested prospective participants share the link to the online questionnaire to other adults (age 18 years or older). Prior to accessing the survey, respondents provided consent to voluntarily participate in the survey. Participants were only allowed to respond once by setting the feature that prevents more than one response, and they were auto-anonymized (without identifying their emails or other contact information). A total of 994 respondents completed the questionnaire; among which 971 were included in the final analysis (23 respondents had missing values and were omitted). The adequacy of sample size is robust enough to perform polytomous IRT models including GRM and GPCM (Hambleton and Swaminathan, 1985; DeMars, 2010).

### Study population

The study population comprised of students, teaching, and non-teaching staffs from a private university in Malaysia and the general population who originated from snowball sampling. Inclusion criteria include age of 18 years and above, and must be a resident of Malaysia during the study period.

### Study instrument

As described in the introduction, the DASS-21 scale asks respondents to answer 21 questions focused on experiencing symptoms of depression, anxiety, and stress in the past week. Participants were given a choice to choose either the DASS-21 Malaysian version or English version based on their ease (Musa, 2017). 90% of the participants used Malaysian version of the DASS-21, and the rest used English version. Participants were provided four response options: 0 = never, 1 = sometimes, 2 = a lot of the time, 3 = most or all of the time (Lovibond and Lovibond, 1995). Total scores for each sub-scale are multiplied by two to interpret scores on the same scale as the DASS-41 (Lovibond and Lovibond, 2020). Higher response values, and higher scores, indicate higher levels of experiencing the condition measured.

### Analytical approach

All analyses were grounded in both the CTT and IRT paradigms, with analyses informed by the Standards for Educational and Psychological Testing (AERA, 2014). Analyses were conducted using R Studio IDE version 1.4. CTT analyses were conducted using the *“psych”* packages (Revelle, 2021). IRT models were fit to polytomous response data using *“mirt”* and *“ltm”* packages (Rizopoulos, 2006; Chalmers, 2021). The R codes used for the analyses can be accessed via the link.

### Classic Test Theory analyses

CTT analyses focused on estimating level of the latent dimension, discrimination, and internal consistency (reliability). Internal consistency was estimated using Cronbach’s alpha. A Cronbach’s alpha of 0.70 or above was considered as good internal consistency (Taber, 2018).

#### Item Response Theory assumptions

Two primary assumptions of IRT are unidimensionality and local independence.

Previous studies have identified a unidimensional structure of DASS-21 response data (Ali and Green, 2019; Lee, 2019); therefore, we assumed unidimensionality and tested this assumption using confirmatory factor analysis (CFA). We ran four CFAs using a Weighted Least Squares Mean and Variance Adjusted (WLSMV) estimator, suitable for ordinal data: one for each sub-scale, and one for the entire DASS-21. Model fit was evaluated using the standardized Root-Mean-Square Error of Approximation (RMSEA), Comparative Fit Index (CFI), Tucker Lewis Index (TLI), and Standardized Root-Mean-Square Residual (SRMR). We considered the cutoffs < = 0.06 for RMSEA and >0.95 for both TLI and CFI proposed by Hu et al., 1999. To test local independence, we used the Q3 statistic to detect violations of local independence and any residual correlation >0.2 above the average correlation (Yen, 1984).

#### Item Response Theory calibration

We assessed the items’ fit using a graded response model (GRM), graded rating scale model (GRSM), and generalized partial credit model (GPCM). These models provide estimates of a slope parameter and three location (step) parameters for each 4-category item. Location parameters in polytomous IRT models indicate the movement between response options. For example, the first location parameter is between responses of 0 to a response of 1.

The most complex of these models is the GRM, which specifies the cumulative probability of a participant’s response (Y) being in or above a category (*k*) as:

$$P\left( \theta \right) = \frac{{e^{a_i\left( {\theta - b_{ij}} \right)}}}{{1 + e^{a_i\left( {\theta - b_{ij}} \right)}}}$$

where *θ* is a respondent’s latent trait ability (i.e., depression, anxiety, or stress), *a*_*i*_ is discrimination for a specific item *i*, and *b*_*ij*_ is the level of the latent dimension (or difficulty parameters) for progressing through each step within an item *j* (e.g., progressing from response category 0 to 1). For each item, the GRM estimates an *a* parameter and three ordered *b*_*j*_ parameters (increasing in the level of the latent dimension). In comparison, the GPCM does not have ordered *b*_*j*_ parameters, and the GRSM does not estimate difficulty for each step (*b*_*ij*_); therefore, the GPCM and GRSM are less computationally complex than the GRM.

All item and person parameters were estimated on a *z*-score scale. Θ was estimated using Expected A-Posteriori (EAP) method (Embretson and Reise, 2000). We compared these models by evaluating the change in the fit using –2loglikelihood, which is distributed as Chi-square with degrees of freedom equal to the difference in the number of parameters for the models, Akaike Information Criterion (AIC), Bayesian Information Criterion (BIC) and Likelihood Ratio Test (LRT). The item fit is assessed using single chi-squared test statistic (S-X2) and RMSEA S-X2 is also used to examine the magnitude of item misfit. After multiple comparison testing, a *p*-value ≤ 0.002 (21 scale items; 0.05/21) indicates potential item misfit (Reeve et al., 2007; Stover et al., 2019)

## Results

### Participants’ characteristics

The participants’ characteristics are shown in Table 1. The samples were predominantly made up of young adults (18 to 25 years: 74%), females (60%), people living in urban areas (68%), and mostly students (51.3%).

**Table 1:** Study participants’ characteristics.

Characteristics	*N* = 971
Age (mean ± S.D.)	23.82 ± 5.71
Sex, *n* (%)	
Males	392 (40.37)
Females	579 (59.62)
Place of residence, *n* (%)	
Rural	310 (31.92)
Urban	661 (68.07)
Age group, *n* (%)	
18 to 20 years	292 (30.1)
21 to 25 years	426 (43.9)
26 to 30 years	168 (17.3)
31 to 40 years	67 (6.9)
41 to 50 years	8 (0.08)
>50 years	10 (0.1)
Occupation, *n* (%)	
Student	498 (51.3)
Full-time employment	321 (33.1)
Part-time employment	43 (4.4)
Unemployed/homemaker	109 (11.2)

*S.D*. standard deviation

### Classical Test Theory (CTT)

Table 2 shows the item response frequency of each item along with CTT statistics. The respondents endorsed all four of the response categories. Over 40% of the respondents agreed that they find it sometimes “hard to wind down”; about half of the participants (47%) had never “experienced breathing difficulty” in the past week; and about 42% of the respondents had never “experienced trembling” in the past week. Item 4 (“I experienced breathing difficulty”) on the anxiety sub-scale, had the least frequently endorsed “most or all of the time” category, with 9% of the total respondents.

**Table 2:** Abbreviated item content, response category percentages, and classical test theory statistics of DASS-21 scale items.

No.	Item	Never (0)	Sometimes (1)	A lot of the time (2)	Most or all of the time (3)	Standardized Inter-item correlation	Inter-item-correlation without the item itself	Mean of the scale if the item is dropped
*Anxiety sub-scale*		(%)						
2	I was aware of dryness of my mouth.	21.42	29.66	24	24.92	0.49	0.44	1.52
4	I experienced breathing difficulty.	47.37	25.95	18.02	8.65	0.66	0.63	0.88
7	I experienced trembling (e.g., in the hands).	42.33	26.88	18.95	11.84	0.69	0.66	1
9	I was worried about situations in which I might panic and make a fool of myself.	21.52	27.7	23.38	27.39	0.79	0.76	1.57
15	I felt I was close to panic.	35.53	30.59	20.91	12.98	0.81	0.78	1.11
19	I was aware of the action of my heart in the absence of physical exertion.	25.23	35.02	23.79	15.96	0.73	0.70	1.30
20	I feel scared without any good reason.	33.16	28.42	19.57	18.85	0.77	0.74	1.24
*Depression sub-scale*		(%)						
3	I couldn’t seem to experience any positive feeling at all.	25.85	37.18	24.51	12.46	0.72	0.69	1.24
5	I find it difficult to work up the initiative to do things.	16.99	33.78	26.78	22.45	0.73	0.69	1.55
10	I felt that I had nothing to look forward to.	28.22	30.59	20.08	21.11	0.77	0.74	1.34
13	I felt down-hearted and blue.	26.47	32.75	22.97	17.82	0.82	0.80	1.32
16	I was unable to become enthusiastic about anything.	30.28	35.74	19.16	14.83	0.79	0.77	1.19
17	I felt I wasn’t worth much as a person.	27.29	27.29	18.64	26.78	0.78	0.75	1.45
21	I feel that life is meaningless.	39.34	22.45	16.48	21.73	0.76	0.73	1.21
*Stress sub-scale*		(%)						
1	I find it hard to wind down.	17.4	40.68	29.25	12.67	0.64	0.61	1.37
6	I tend to over-react to situations.	20.19	36.66	23.69	19.46	0.72	0.69	1.42
8	I feel that I was using a lot of nervous energy.	25.54	30.07	23.48	20.91	0.78	0.76	1.40
11	I find myself getting agitated.	27.7	34.29	23.27	14.73	0.82	0.80	1.25
12	I find it is difficult to relax.	27.6	37.49	21.11	13.8	0.80	0.78	1.21
14	I was intolerant of anything that kept me from getting on with what I was doing.	26.26	36.77	24.51	12.46	0.77	0.74	1.23
18	I feel that I was rather touchy.	25.85	32.85	23.69	17.61	0.68	0.65	1.33

#### Reliability analysis

Overall, Cronbach’s alpha of the DASS-21 was 0.959, which indicates an excellent internal consistency. The Cronbach’s alpha value of each sub-scale for anxiety, depression, and stress were 0.87 (95% CI 0.86 to 0.89), 0.92 (95% CI 0.91 to 0.93) and 0.89 (95% CI 0.88 to 0.90), respectively. Alpha for these data would remain consistent if items were deleted, staying within the 95% confidence interval range of 0.95 to 0.97.

The average inter-item correlation was 0.739, and corrected inter-item correlations for each of the sub-scales indicated acceptable discrimination (0.69–0.80, depression; 0.44–0.78, anxiety; 0.61–0.80, stress). Item 2 (“I was aware of dryness of my mouth.”), on the anxiety sub-scale, was the least discriminating item (0.49); Item 11 (“I find myself getting agitated.”), on the stress sub-scale, had the highest item discrimination (0.82).

### Testing assumptions

#### Unidimensionality

CFA confirmed the one-dimensional structure of the overall DASS-21 scale: CFI of 0.90, TLI of 0.89, RMSEA of 0.088 (90% CI 0.084 to 0.092) and SRMR of 0.043 suggested that the model fit the data (Hooper et al., 2008). The assumption of unidimensionality for the anxiety and stress sub-scales were also empirically supported by CFI, TLI, RMSEA, and SRMR (see Table 3). The depression sub-scale, however, had worse RMSEA (0.112; 90% CI 0.098 to 0.127) but acceptable CFI and TLI.

**Table 3:** Examining the dimensionality of DASS-21 scale and its sub-scales using confirmatory factor analysis.

	CFI	TLI	RMSEA	SRMR
Depression	0.961	0.942	0.112 (90% C.I. 0.098–0.127)	0.033
Anxiety	0.979	0.968	0.069 (90% C.I. 0.054–0.084)	0.026
Stress	0.982	0.973	0.067 (90% C.I. 0.052–0.082)	0.023
Overall DASS-21	0.900	0.889	0.088 (90% C.I 0.084–0.092)	0.043

*RMSEA* standardized root-mean-square error of approximation, *CFI* Comparative Fit Index, *TLI* Tucker Lewis Index, *SRMR* standardized root-mean-square residual.

#### Local independence

The effect size of the model fit (MADaQ3) and test of global model fit (max aQ3) statistics, and SRMR and Standardized Root-Mean-Square Root of Squared Residuals (SRMSR) were estimated for assessing the local independence (Liu and Maydeu-Olivares, 2012; Kline, 2016). MADaQ3 and max aQ3 were 0.0851 and 0.4388, respectively; SRMR and SRMSR were 0.081 and 0.101, respectively, supporting the assumption of local independence.

### Item Response Theory (IRT) calibration

#### Item properties

**Fig. 1:**
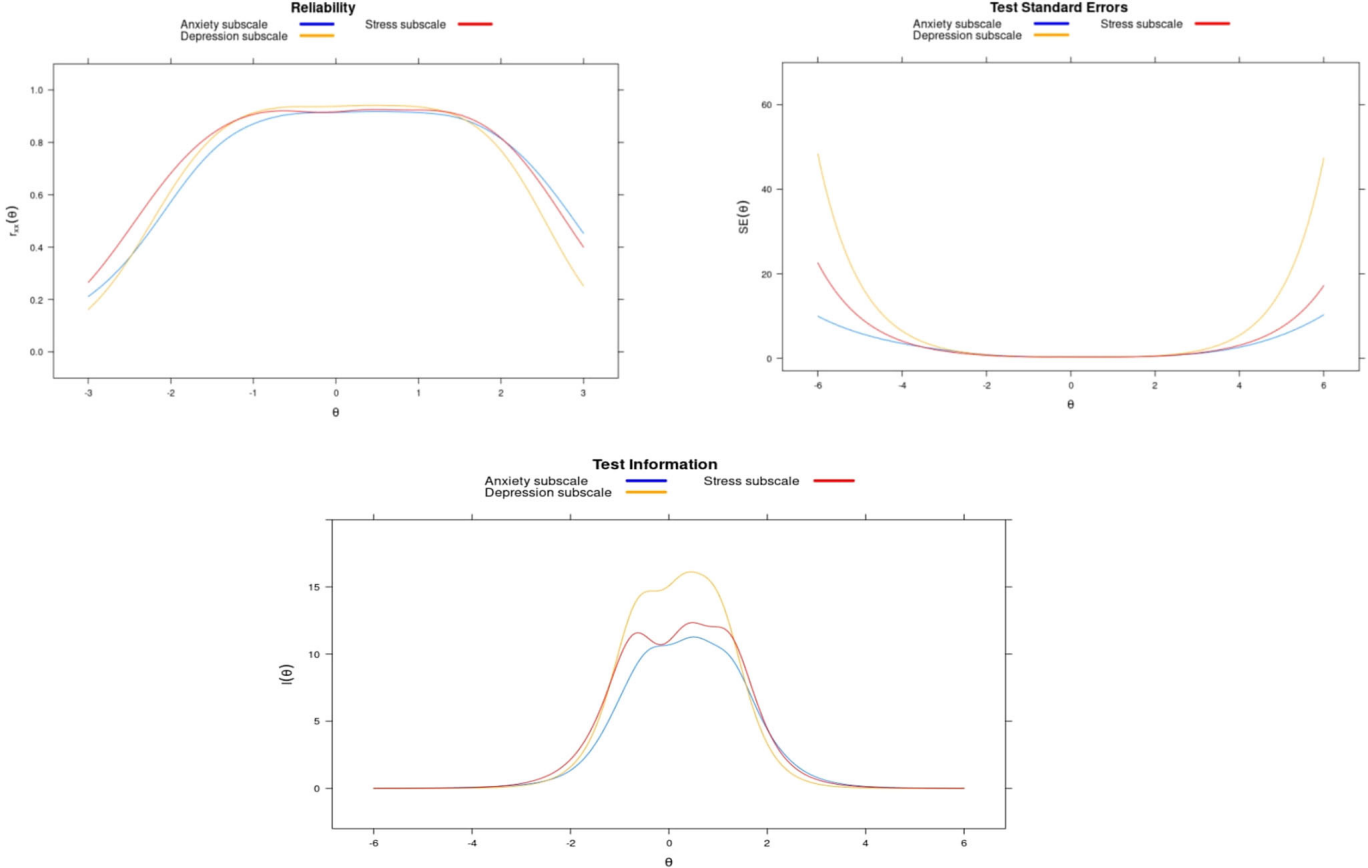
Test reliability, standard errors, and information function of the DASS-21 subscales (depression, anxiety, and stress subscales are indicated with yellow, blue, and red lines, respectively).

GRM had better empirical fit to sub-scale response data when considering AIC, BIC, –2loglikelihood values, and the LRT (see Supplementary Tables 1–4). Therefore, the GRM was used to calibrate and examine item-level parameters for each of the sub-scales. In addition, we assessed the performance of the GRM, GRSM, GPCM, and the Partial Credit Model (PCM; a Rasch model) for the overall DASS-21. The GRM also had the best model fit for the overall DASS-21 response data. DASS-21 sub-scale item characteristics are displayed in Table 4. Standard error of measurement for each of the items is minimized in the theta range of −3.0 to 3.0, with the anxiety sub-scale having the best reliability across the entire range of theta. Test reliability, standard error, and information functions for each sub-scale are provided in Fig. 1. Individual item function, Item Categorical Response Curves, and Operation Characteristic Curves for each sub-scale are shown in Supplemental Figs. 1–9.

**Table 4:** DASS-21 and sub-scale item characteristics under graded response model.

	Item parameters				Item fit statistics		
Item no.	*a*	b1	b2	b3	S_X2	RMSEA	*p*-value
*Depression sub-scale*							
3	2.24	−0.81	0.41	1.48	48.93	0.02	0.06
5	1.95	−1.27	0.02	1.01	63.11	0.03	0.01
10	3.11	−0.66	0.24	0.92	36.49	0.01	0.35
13	2.90	−0.74	0.27	1.09	37.60	0.01	0.27
16	3.08	−0.60	0.46	1.21	34.18	0.01	0.27
17	2.96	−0.70	0.12	0.71	54.63	0.03	0.01
21	3.33	−0.32	0.32	0.88	53.48	0.03	0.01
*Anxiety sub-scale*							
2	1.04	−1.51	0.06	1.30	62.86	0.02	0.02
4	2.00	−0.08	0.81	1.80	43.22	0.01	0.19
7	2.17	−0.25	0.63	1.50	47.89	0.02	0.11
9	2.69	−0.93	−0.01	0.72	32.11	0.00	0.46
15	3.39	−0.41	0.48	1.27	21.23	0.00	0.85
19	2.11	−0.84	0.38	1.30	61.79	0.03	0.01
20	2.45	−0.52	0.37	1.08	56.68	0.03	0.01
*Stress sub-scale*							
1	1.62	−1.35	0.29	1.67	51.88	0.02	0.04
6	2.18	−1.08	0.20	1.09	47.09	0.02	0.08
8	2.45	−0.81	0.18	0.99	26.53	0.00	0.78
11	3.28	−0.67	0.36	1.18	34.92	0.02	0.21
12	3.22	−0.67	0.45	1.23	34.45	0.02	0.19
14	2.56	−0.75	0.42	1.39	35.97	0.01	0.25
18	1.74	−0.89	0.31	1.29	60.70	0.03	0.01
*Overall DASS-21*							
1	1.63	−1.34	0.29	1.65	138.23	0.02	0.04
2	0.95	−1.61	0.06	1.38	195.87	0.02	0.00
3	2.07	−0.83	0.44	1.51	95.52	0.00	0.66
4	1.71	−0.10	0.86	1.93	128.47	0.01	0.12
5	2.02	−1.25	0.04	1.00	135.12	0.02	0.03
6	2.00	−1.11	0.22	1.12	125.95	0.01	0.10
7	1.82	−0.27	0.66	1.61	159.50	0.02	0.00
8	2.50	−0.80	0.17	0.98	101.91	0.01	0.35
9	2.60	−0.93	−0.02	0.72	105.52	0.01	0.22
10	2.44	−0.70	0.27	0.98	129.70	0.02	0.03
11	3.15	−0.66	0.37	1.19	98.98	0.02	0.09
12	2.98	−0.67	0.46	1.24	96.09	0.01	0.15
13	3.10	−0.71	0.29	1.05	79.55	0.00	0.49
14	2.62	−0.73	0.42	1.38	134.32	0.02	0.00
15	2.94	−0.42	0.48	1.29	116.91	0.02	0.02
16	2.83	−0.59	0.50	1.23	141.52	0.03	0.00
17	2.48	−0.72	0.15	0.76	126.24	0.02	0.04
18	1.82	−0.87	0.31	1.26	138.40	0.01	0.09
19	2.23	−0.81	0.36	1.25	115.16	0.01	0.14
20	2.52	−0.52	0.36	1.05	113.18	0.01	0.19
21	2.49	−0.32	0.36	0.94	123.83	0.01	0.09

*Note*. *a*—discrimination parameter; b1, b2, and b3—difficulty parameters, RMSEA—root-mean-square error of approximation and *p*-value.

On the depression sub-scale, item information was maximized for Item 21 (“life is meaningless”), Item 10 (“nothing to look forward to”), and Item 16 (“unable to become enthusiastic about anything”), while Item 5 (“difficult to work up the initiative”) provided the least amount of information. Item 5 also had the lowest mean threshold, indicating that people with lower levels of the latent trait of depression had higher probability of endorsing the item. Items with higher mean thresholds—Item 16 and Item 3 (“couldn’t experience any positive feeling”)—were more related to the symptoms of feeling pleasure.

The anxiety sub-scale had one item with item discrimination over 3.0: Item 15 (“close to panic”), and the item providing less information was Item 2 (“dryness in mouth”). The lowest mean thresholds on the anxiety sub-scale were observed for Item 9 (“worried about situations I might panic”) and Item 2. Higher mean thresholds were indicated for items with stronger physiological symptoms of anxiety: Item 4 (“difficulty breathing”), Item 7 (“trembling”), and Item 15.

The stress sub-scale had two items with relatively larger item information functions: Item 11 (“getting agitated”) and Item 12 (“difficult to relax”). Item 6 (“tend to overreact”) and Item 8 (“nervous energy”) had the lowest mean thresholds. Item 14 (“intolerant of anything that kept me from getting on”) and Item 12 had the highest mean thresholds.

#### Person properties

Person fit estimates indicated the majority (>95%) of participants within each sub-scale had Zh statistics between the range of −1.96 and 1.96, indicating strong person fit. Latent scores (*θ*) indicated the participants had latent scores between −6.309 to 3.035 with a mean of 0.174 (see Table 5). Raw summated and latent (IRT estimated) scores of the Depression, Anxiety, and Stress sub-scales, and overall DASS-21 were strongly correlated (*r*s = 0.985, 0.978, 0.985, and 0.980, respectively).

**Table 5:** Person fit estimates of DASS-21 and its sub-scale.

Scale	Mean (range)	Percent of participants with good person fit^a^
DASS-21 overall scale	0.174 (−6.309 to 3.035)	85%
Depression	0.276 (−4.322 to 1.865)	95.2%
Anxiety	0.309 (−3.940 to 1.768)	97.8%
Stress	0.283 (−5.625 to 1.888)	95.0%

^a^Good person fit was determined by Zh statistics between −1.96 and 1.96.

## Discussion

Mental health conditions are among the most common disease burdens worldwide, and they are especially prevalent in Southeast Asia (including Malaysia). Accurately measuring the most common mental health symptoms and conditions—depression, anxiety, and stress—is imperative for clinical treatment, public health programming, and research. Therefore, the present study aimed to assess the psychometric properties of scores elicited from the DASS-21 among a convenience sample of Malaysians during the COVID-19 pandemic. We applied CTT and IRT methods to assess item and test properties including the level of the latent dimension (difficulty), discrimination, reliability, and dimensionality.

CFA analyses provided empirical support for the IRT assumption of unidimensionality for each of the sub-scales and the overall DASS-21. However, model fit indices for the depression sub-scale underperformed. This could be due to a variety of reasons including differences in participant response processes (e.g., understanding of and response to the item), or different dialects used across Malaysian states. For example, a previous study conducted in western part of the Malaysia highlighted the limitation that the current Malaysian version may underperform in eastern parts where they have different dialects (Musa et al., 2007) and some studies have questioned the interpretation of item wording across southeast Asian cultures (Oei et al., 2013). Therefore, future research should be conducted to better understand the dialect-based appropriateness of DASS-21 items among different regions of Malaysians to ensure that items are adequately measuring normative perceptions of depression, anxiety, and stress.

In both CTT and IRT analyses, item statistics (e.g., level of the latent dimension and discrimination) varied. While CTT analyses can be useful for understanding properties of an entire test, results from these analyses are (unlike IRT analyses) sample dependent. Results from our CTT analyses indicate that scores elicited from the DASS-21 sub-scales had strong item discrimination and excellent internal consistency reliability within our sample. To illustrate that these properties are sample dependent, internal consistency within our study for the depression and anxiety scales (0.92 and 0.87, respectively) were higher than estimates from other Malaysian samples (0.82 and 0.76, respectively) (Oei et al., 2013). The overall Cronbach’s alpha for the entire DASS-21 responses was 0.96; if items were deleted, alpha would stay within the range of 0.95 to 0.97.

A GRM best fit the response data for each of the sub-scales, and of the overall scale—indicating that each item had a unique discrimination and mean threshold (level of the latent dimension) parameter. Across the three sub-scales, the most discriminating items were “*I feel that life is meaningless*” (depression), “*I felt I was close to panic*” (anxiety), and “*I find myself getting agitated”* (stress). An additional measure elicited from IRT estimates are item mean threshold, a measure of level of the latent dimension. Items with higher mean threshold, require a person have higher latent trait scores (in our case, worse depression, anxiety, or stress) to endorse higher response options. The three items with the highest mean threshold were “*I could not seem to experience any positive feeling at all*” (depression), “*I experiencing breathing difficulty*” (anxiety), and “*I was intolerant of anything that kept me from getting on with what I was doing*” (stress). These items, with high discrimination and high mean threshold, relate to more severe symptomology of mental health conditions.

Overall, scores (on a *z*-score scale) on the depression, anxiety, and stress sub-scales indicated low levels of these mental health issues (person-means = 0.276, 0.309, and 0.283, respectively); however, these scores indicated slightly increased symptom endorsement than the expected score of 0. This is not assumed to be an artifact of the DASS-21, as the DASS-21 has consistent measurement properties and scoring with other depression screeners (e.g., the Patient Health Questionnaire) (Peters et al., 2021). The largest range among scores was observed on the stress sub-scale, with person scores ranging from 5.5 standard deviations below the average stress score to over 1.9 standard deviations above the average stress score. Similar to CTT analyses, IRT findings supported the notion of strong reliability of scores across the range of theta between –3.0 and 3.0, with more measurement error beyond this range.

### Limitations

Results from this study should be interpreted with respect to the limitations of the study design. The use of convenience, non-representative sample of Malaysians may weaken the inferences and stability of IRT item properties. However, IRT person estimates include a breadth of scores providing accurate samples of the IRT model and parameter calibration. The COVID-19 pandemic situation could have impacted mental health status of the participants overall, but we believe the DASS-21 would have still provided information about item functioning and this could be an example scenario where the scale needs to perform well by identifying people who are in the poor mental health states. The depression sub-scale had worse model fit in CFA (unidimensionality) estimates; although unidimensionality was supported—through theory, CFI, TLI, and local independence statistics—future research should seek to identify qualitative issues with model misfit with respect to the depression sub-scale. An additional limitation is the use of both Malay and English versions of the questionnaire; due to a smaller number of the individuals who utilized English version (10%), we were not able to estimate differences across these two groups. Lastly, the lack of coverage on the original survey of other mental health screening instruments, to provide additional evidence of convergent validity.

## Conclusions

The DASS-21 instrument is frequently used in mental health research and practice, particularly in Malaysia (Shamsuddin et al., 2013; Wong et al., 2021). Therefore, understanding the psychometric properties of response data elicited from the DASS-21 is crucial to ensuring that research conducted with this scale is valid. Findings from this study provide evidence of validity supporting the use of the DASS-21 as a mental health screening tool among Malaysians. Specifically, standard error of measurement was minimized (and reliability, maximized) across a range of theta between 3 standard deviations below and above the average depress, anxiety, and stress level; this provides strong evidence of potential utility in identifying participants who are and are not experiencing these mental health issues. Further, the strong correlation between summated scores (used in practice) and latent scores provides additional support for the use of this scale in practice—where latent scores are frequently unavailable. Additional research is necessary to assess the presence of Differential Item Functioning among Malaysian samples, comprising both eastern and western parts in ensuring that test content is dialect sensitive and accurately measuring cultural norms of depression, anxiety, and stress.

## Supplementary information

Supplementary file

## Data Availability

All data files used in the study are available from the Harvard Dataverse database via 10.7910/DVN/AJVLN6 and the corresponding analysis codes are available from the Open Software Foundation, the centre for Open Science via osf.io/d4kar
