# Supplementary material for: Psychometric properties of the 21-item Depression, Anxiety, and Stress Scale (DASS-21) among Malaysians during COVID-19: a methodological study
Source: Humanit Soc Sci Commun. 2022 Jun 29;9(1):220. doi: 10.1057/s41599-022-01229-x (PMC9244484; doi:10.1057/s41599-022-01229-x)

**Supplementary file to**: **Psychometric Properties of the 21-item Depression, Anxiety, and Stress Scale (DASS-21) among Malaysians during COVID-19: A Methodological Study**

Arulmani Thiyagarajan^1^, Tyler G James^2^ and Roy Rillera Marzo^3, 4, 5^

^1^Department of Clinical Epidemiology, Leibniz Institute for Prevention Research and Epidemiology - BIPS, Germany

^2^Department of Family Medicine, University of Michigan, USA

^3^Department of Community Medicine, International Medical School, Management and Science University, Malaysia

^4^Department of Community Medicine, Faculty of Medicine, Asia Metropolitan University, Malaysia

^5^Global Public Health Jeffrey Cheah School of Medicine and Health Sciences, Monash University, Malaysia

**Supplementary table 1:** Overall DASS-21 item properties using AIC, BIC, -2loglikelihood

**Supplementary table 2:** Anxiety subscale item properties using AIC, BIC, -2loglikelihood

**Supplementary table 3:** Depression subscale item properties using AIC, BIC, -2loglikelihood

**Supplementary table 4:** Stress subscale item properties using AIC, BIC, -2loglikelihood

**Supplemental Figure 1:** DASS-21 depression sub-scale item information function under the Graded Response Model.

**Supplemental Figure 2:** DASS-21 depression sub-scale Categorical Response under the Graded Response Model.

**Supplemental Figure 3:** DASS-21 depression sub-scale Operation Characteristic Curves under the Graded Response Model.

**Supplemental Figure 4:** DASS-21 anxiety sub-scale item information function under the Graded Response Model.

**Supplemental Figure 5:** DASS-21 anxiety sub-scale Categorical Response under the Graded Response Model.

**Supplemental Figure 6:** DASS-21 anxiety sub-scale Operation Characteristic Curves under the Graded Response Model.

**Supplemental Figure 7:** DASS-21 stress sub-scale item information function under the Graded Response Model.

**Supplemental Figure 8:** DASS-21 stress sub-scale Categorical Response under the Graded Response Model.

**Supplemental Figure 9:** DASS-21 stress sub-scale Operation Characteristic Curves under the Graded Response Model.

**Supplementary table 1**: Overall DASS-21 item properties using AIC, BIC, -2loglikelihood

| **Model type** | **Akaike Information Criterion (AIC)** | **Bayesian Information Criterion (BIC)** | **-2 logLikelihood** |
| --- | --- | --- | --- |
| *Graded Response Model (GRM)* | 42015.21 | 42424.99 | -20923.61 |
| *Graded Rating Scale Model (GRSM)* | 42461.78 | 42676.43 | -21186.89 |
| *Generalized Partial Credit Model (GPCM)* | 42262.09 | 42671.87 | -21047.04 |
| *Partial Credit Model (PCM)* | 43170.5 | 43477.83 | -21522.25 |

**Supplementary table 2:** Anxiety subscale item properties using AIC, BIC, -2loglikelihood

| Model type | Akaike Information Criterion (AIC) | Bayesian Information Criterion (BIC) | -2 logLikelihood |
| --- | --- | --- | --- |
| *Graded Response Model (GRM)* | 15190 | 15326 | -7566.8 |
| *Graded Rating Scale Model (GRSM)* | 15311 | 15389 | -7639.4 |
| *Generalized Partial Credit Model (GPCM)* | 15256 | 15392 | -7599.9 |

**Supplementary table 3:** Depression subscale item properties using AIC, BIC, -2loglikelihood

| Model type | Akaike Information Criterion (AIC) | Bayesian Information Criterion (BIC) | -2 logLikelihood |
| --- | --- | --- | --- |
| *Graded Response Model (GRM)* | 14302 | 14439 | -7123.1 |
| *Graded Rating Scale Model (GRSM)* | 14320 | 14398 | -7143.9 |
| *Generalized Partial Credit Model (GPCM)* | 14377 | 14514 | -7160.5 |

**Supplementary table 4:** Stress subscale item properties using AIC, BIC, -2loglikelihood

| **Model type** | **Akaike Information Criterion (AIC)** | **Bayesian Information Criterion (BIC)** | **-2 logLikelihood** |
| --- | --- | --- | --- |
| *Graded Response Model (GRM)* | 14879 | 15016 | -7411.7 |
| *Graded Rating Scale Model (GRSM)* | 14951 | 15029 | -7459.6 |
| *Generalized Partial Credit Model (GPCM)* | 14981 | 15117 | -7462.5 |

**Supplemental figure 1:** DASS-21 depression sub-scale item information function under the graded response model.


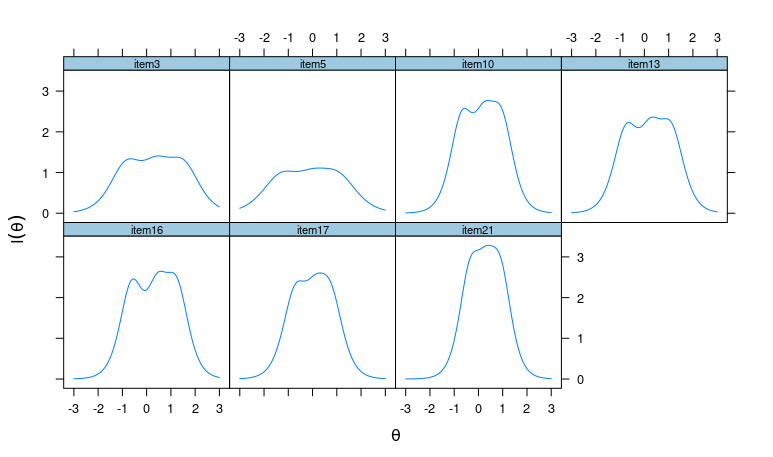


**Supplemental figure 2:** DASS-21 depression sub-scale categorical response under the graded response model.


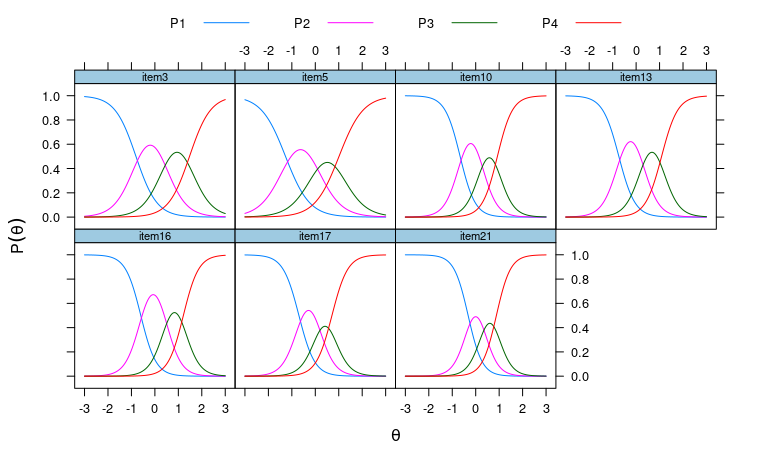


**Supplemental figure 3:** DASS-21 depression sub-scale operation characteristic curves under the graded response model.


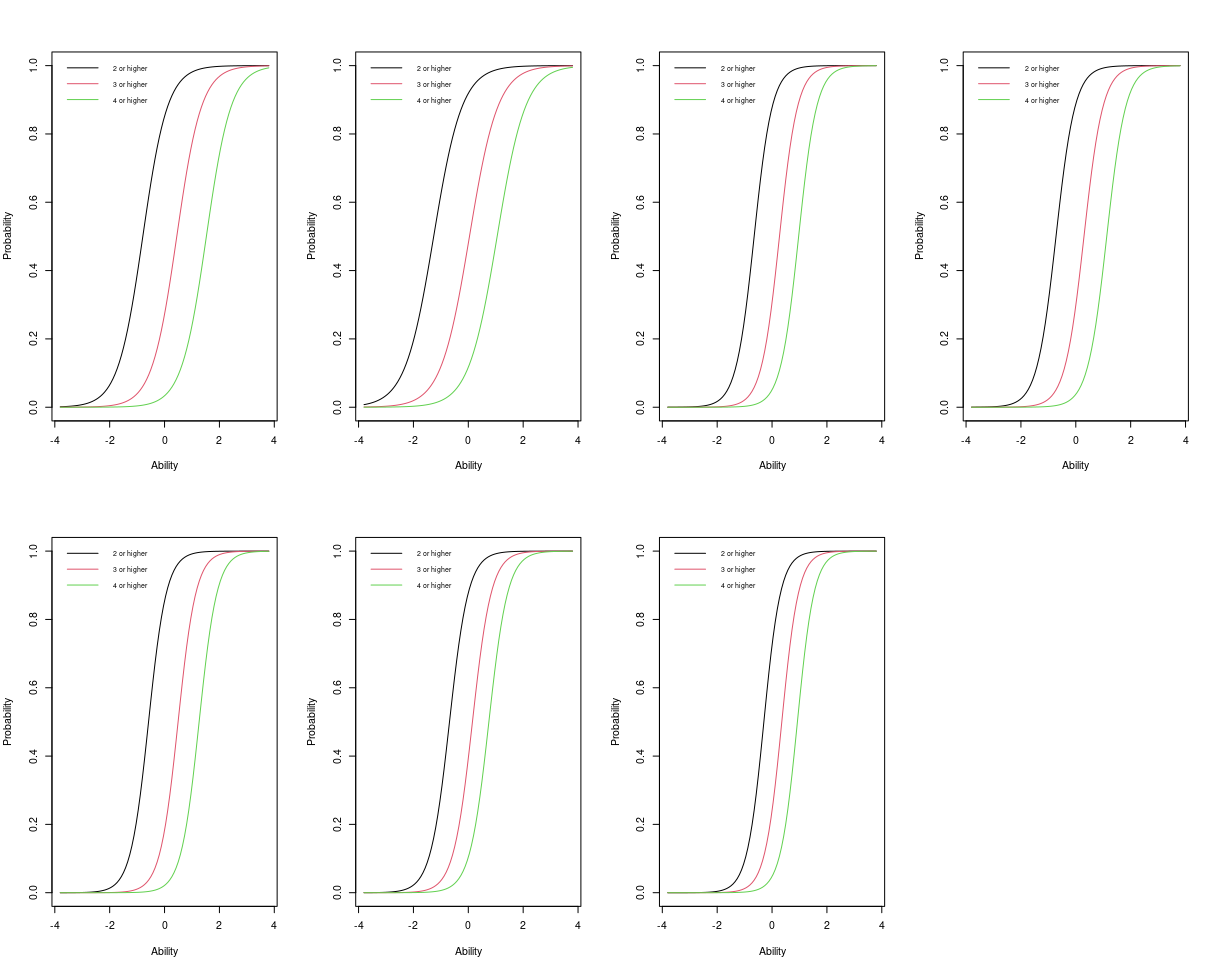


**Supplemental figure 4:** DASS-21 anxiety sub-scale item information function under the graded response model.
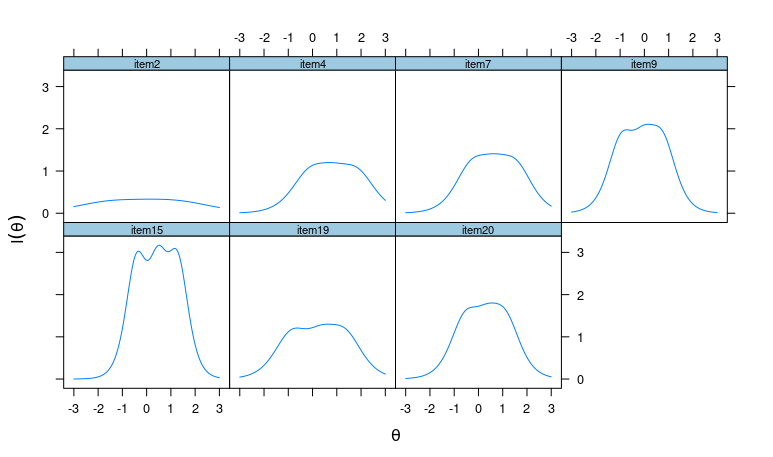


**Supplemental figure 5:** DASS-21 anxiety sub-scale categorical response under the graded response model.


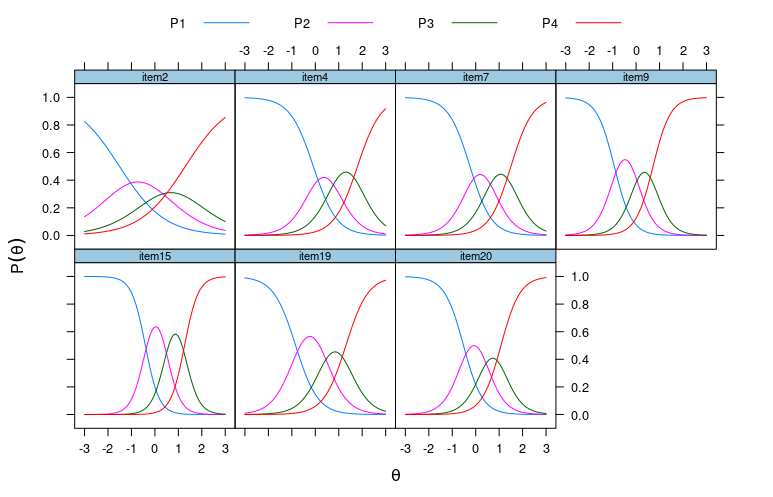


**Supplemental figure 6:** DASS-21 anxiety sub-scale operation characteristic curves under the graded response model.


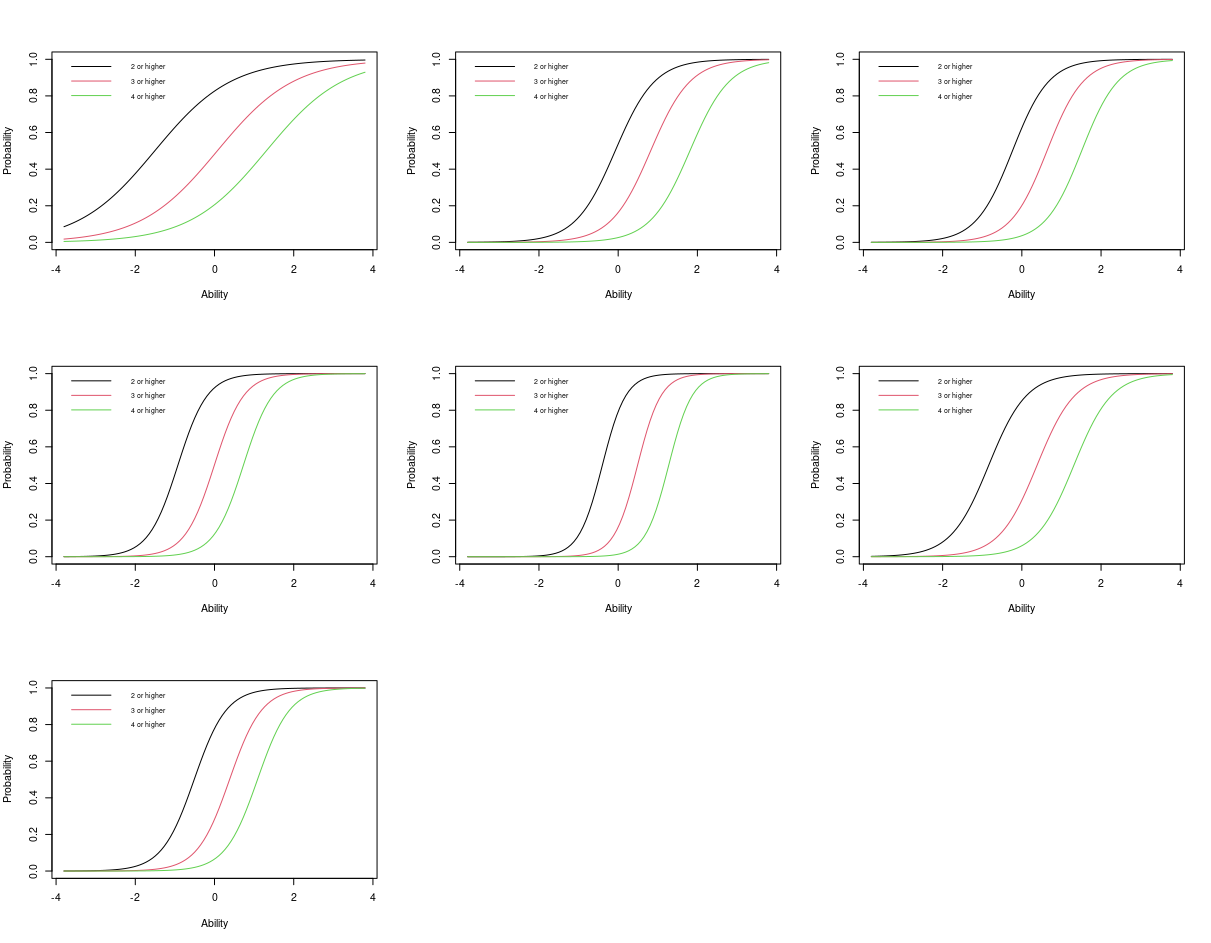


**Supplemental figure 7**: DASS-21 stress sub-scale item information function under the graded response model.


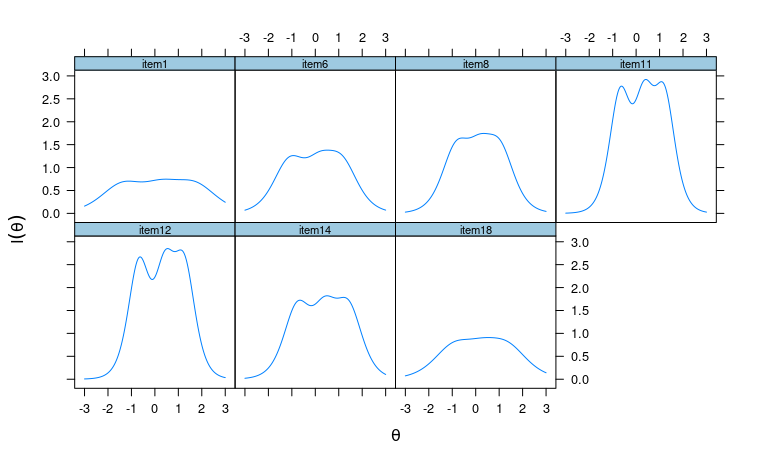


**Supplemental figure 8:** DASS-21 stress sub-scale categorical response under the graded response model.


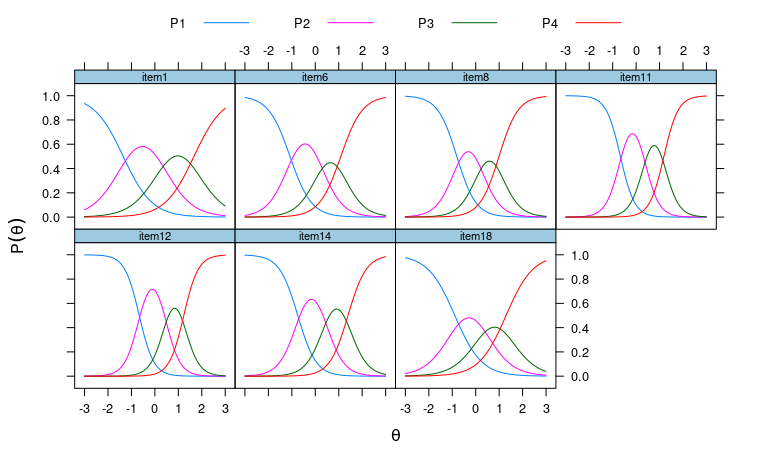


**Supplemental figure 9:** DASS-21 stress sub-scale operation characteristic curves under the graded response model.


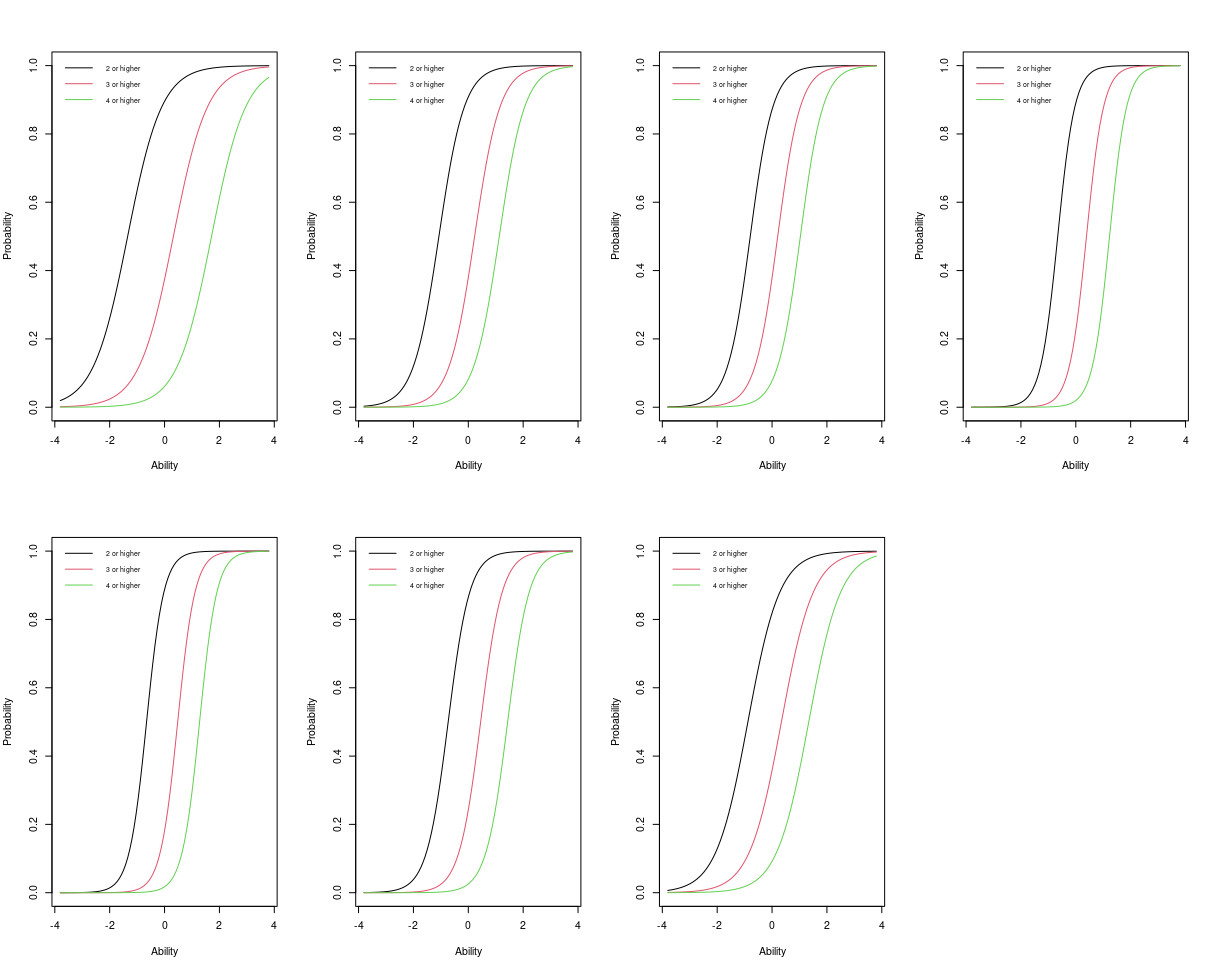

Supplement: Supplementary file 1 — Supplementary file [file 41599_2022_1229_MOESM1_ESM.docx]
